# Immunological and clinical consequences of splenectomy in a multiple sclerosis patient treated with natalizumab

**DOI:** 10.1186/1742-2094-10-123

**Published:** 2013-10-09

**Authors:** De-Hyung Lee, Anne Waschbisch, Alexandra B Lämmer, Arnd Doerfler, Stefan Schwab, Ralf A Linker

**Affiliations:** 1 Department of Neurology, Friedrich Alexander University of Erlangen-Nuremberg, Schwabachanlage 6, Erlangen 91054, Germany; 2 Department of Neuroradiology, Friedrich Alexander University of Erlangen-Nuremberg, Schwabachanlage 6, Erlangen 91054, Germany

**Keywords:** Progressive multifocal leukoencephalopathy, Multiple sclerosis, Natalizumab, Splenectomy, CD19 positive B cells

## Abstract

**Objective:**

Here we report a case of a splenectomized white woman with natalizumab-associated progressive multifocal leukoencephalopathy (PML), occurring as early as after 11 infusions and provide blood fluorescence-activated cell sorting (FACS) analyses before and after natalizumab treatment.

**Design:**

This is a report of a single case with immunological studies.

**Methods:**

Methods comprised neurologic examination, magnetic resonance imaging, and cerebrospinal fluid (CSF) studies as well as immune cell FACS analyses from blood.

**Results:**

Diagnosis of PML was established after positive John Cunningham virus (JCV) DNA was detected in the CSF. An immune reconstitution inflammatory syndrome was treated with repeated cycles of steroid pulses and intravenous immunoglobulins. Reduced numbers of memory B cells, which might play an important role in antiviral immune response, were detected in the blood. Moreover the percentage of CD19+ B cells was elevated in our post-splenectomy patient as compared to a control cohort of multiple sclerosis (MS) patients under natalizumab therapy.

**Conclusion:**

Splenectomy may increase the risk for the development of natalizumab-associated PML via effects on the B cell compartment. It may be regarded as a risk factor in MS patients independent from the duration of disease.

## Background

Before the era of natalizumab as first monoclonal antibody in multiple sclerosis (MS) therapy arose, progressive multifocal leukoencephalopathy (PML), which is caused by the John Cunningham polyoma virus (JCV), was more or less exclusively seen in immunocompromised individuals, especially those suffering from human immunodeficiency virus (HIV) infection [1,2]. Natalizumab is a highly effective treatment option for relapsing remitting MS with positive data from the pivotal phase III trial yielding a reduction in annual relapse rate by 68% and a reduction in disability progression over 42% *versus* placebo [3]. Yet, a rare but potentially severe adverse event of natalizumab is the development of PML. At present, there are more than 350 cases of natalizumab-associated PML reported worldwide, with an overall incidence of 2.5/1,000. Treatment with unselective immunosuppressants displays an independent epidemiologic risk factor for the development of PML, especially after 24 months of treatment duration [4]. These data suggest that the probability to develop a PML might be associated with an impaired integrity of the immune system. To date, the exact immune factors contributing to an increased PML susceptibility are still not clearly defined. In particular, there are no data on the role of the spleen for immune responses against JC virus in MS patients. The spleen plays a key role in the homeostasis of the immune system. By linking the innate and adaptive immune system, it orchestrates the immune defense that protects from infections [5]. In splenectomized individuals, absolute lymphocyte counts are persistently elevated due to an increase in the absolute CD4, CD8, B cell, and natural killer (NK) cell numbers [6]. Similar changes can be observed in natalizumab-treated patients [7,8]. Moreover, natalizumab treatment may elicit prominent effects on the composition of the circulating B cell populations. In particular circulating B cells and especially pre-B cells are most prominently elevated among the immune cell subsets [9-11]. Likewise dominant changes on the peripheral blood B cell compartment have also been observed in splenectomized patients. Natalizumab associated changes have been attributed to the mobilization of hematopoetic precursor cells from the bone marrow and a redistribution of cells due to the inhibition of leukocyte migration and homing to secondary lymphoid organs [12-14]. Splenectomy and natalizumab may thereby have synergistic effects on the immune cell composition of the peripheral blood thus increasing the risk for PML.

## Methods

Peripheral blood mononuclear cells (PBMC) were isolated by centrifugation on a Lymphoprep™ (Fresenius Kabi Norge AS, Oslo, Norway) density gradient. To allow comparative longitudinal analysis, PBMC were immediately cryopreserved. Blood from our PML patient had been drawn 22 months before the primary diagnosis of PML. At that time the patient was not treated with disease modifying drugs (T_0_). The second blood sample (T_1_) was drawn when the patient was hospitalized for PML, the third sample (T_2_) was obtained 21 days later. Results of our splenectomized patients were compared with a control cohort of MS patients under natalizumab therapy (mean age, 34.4 years; range, 24-43 years; female to male ratio, 3.5:1; mean disease duration, 7.8 years; range, 2–16 years; mean expanded disability status scale (EDSS), 2.95; range, 1.5-5.5). After gentle thawing, cells were washed twice in phosphate buffered saline (PBS) containing 0.1% sodium azide and 1% bovine serum albumine, followed by Fc receptor blocking with human IgG (Sigma-Aldrich, Munich, Germany). Afterwards, cells were incubated for 30 min with specific monoclonal antibodies. The B cell gating strategy of fluorescence-activated cell sorting (FACS) analysis has been provided as Additional file 1: Figure S1. The following anti-human monoclonal antibodies (clone) and the respective isotype control antibodies were used (all fluorochrome-conjugated): anti-CD19 FITC (HIB19, BD Biosciences), anti-CD27 efluor®450 (O323, ebioscience), anti-IgM PE (SA-DA4, ebioscience), anti-IgD PerCP-CY5.5 (IA6-2, Biolegend). Cells were analyzed on a FACSCanto™ II using the FACSDiva™ Software (BD Biosciences, Heidelberg, Germany).

## Case presentation

**Figure 1:**
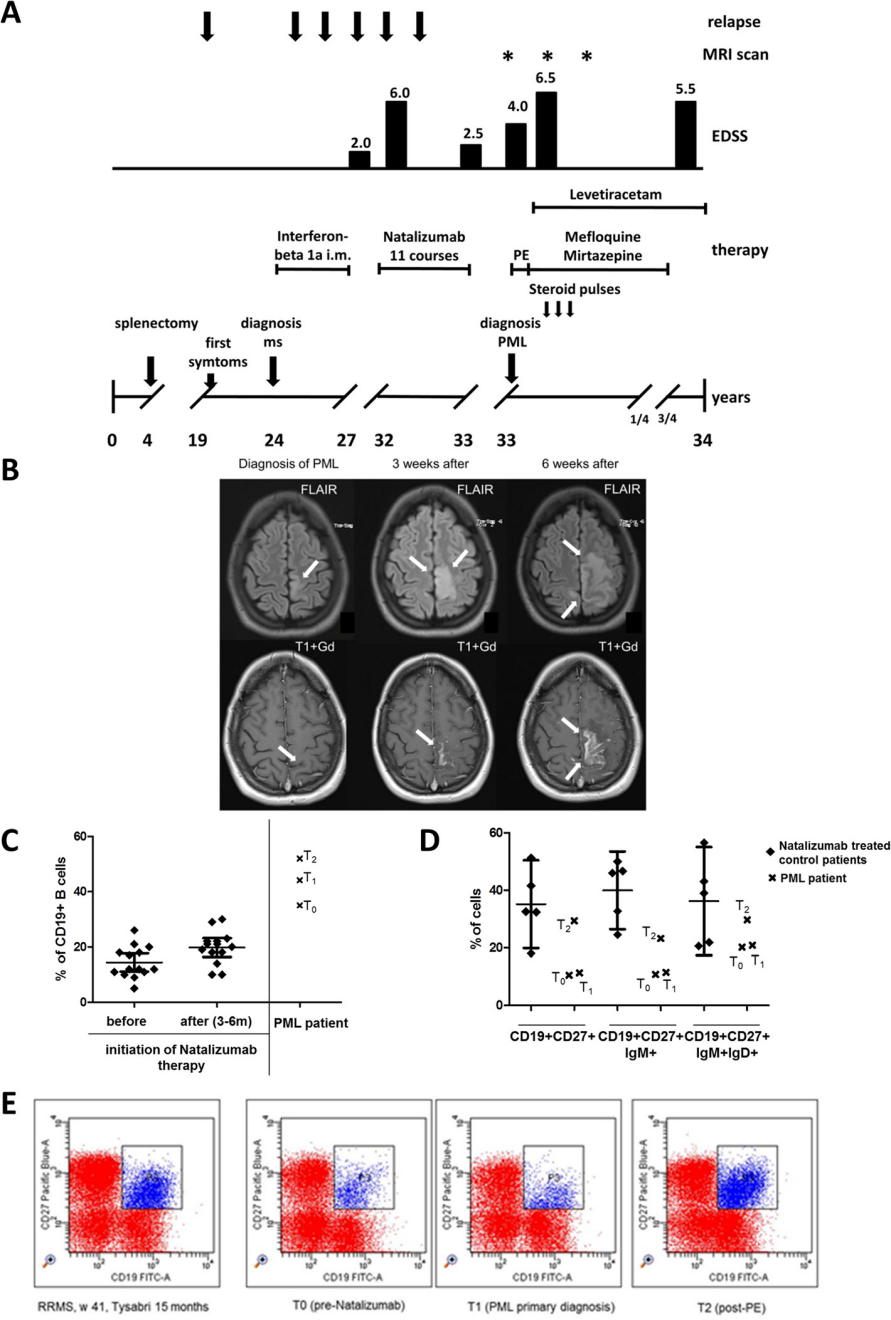
**Frequency of B cells and B cell subgroups before and after natalizumab treatment. (A)** Schematic overview of the clinical course of MS and PML in our splenectomized patients**.** PE, plasma exchange. **(B)** MRI over the course of disease. On admission, an axial flair-attenuated inversion recovery (FLAIR)-weighted scan of the brain displayed signal hyperintense lesion in the frontal- parietal lobe (arrow), 3 weeks after diagnosis lesion volume increased in the course of the IRIS, 6 weeks after diagnosis nearly the whole frontal- parietal lobe is affected (arrow). T1-weighted images with Gd displayed a very faint Gd enhancement (arrow), 3 weeks after diagnosis and 6 weeks after diagnosis lesion volume with Gd enhancement increased (arrow). **(C)** The percentage of CD19+ cells within the lymphocyte gate was assessed by flow cytometry in relapsing-remitting MS (RRMS) patients with active disease before and 3 to 6 months after initiation of Natalizumab therapy (*n* = 14) and in the splenectomized patient. Different time points marked by a cross; T_0_, before natalizumab therapy; T_1_, at the time of PML; T_2_, 3 weeks after PE. **(D)** The percentages of specialized B cell subpopulations (CD19 + CD27+ memory cells, IgM + memory cells, and IgM + IgD + marginal cell-like B cells) were assessed by flow cytometry in natalizumab-treated RRMS control patients (*n* = 5; mean + 95% CI) and the splenectomized PML patient at different time points marked by a cross. T_0_, before natalizumab therapy; T_1_, at the time of PML; T_2_, 3 weeks after PE. **(E)** Dot blot of CD19 + CD27+ memory B cells within the lymphocyte gate in the splenectomized patient and a representative, natalizumab-treated RRMS control.

At the age of 4 years, a now 34–year-old white woman underwent splenectomy after a neonatal omphalitis with portal vein thrombosis and esophageal varices. The incidence of common infections during child- as well as adulthood was not increased in comparison to healthy persons indicating a generally intact immunological response. At the age of 19 years, the patient noticed sensory disturbances in the feet with unsteadiness both of which resolved spontaneously. Five years later she was admitted to an outside hospital due to a hemiparesis of the left side where the diagnosis of a MS was established. Magnetic resonance imaging (MRI) of the brain revealed several periventricular lesions as well a gadolinium-enhancing lesion. Analysis of cerebrospinal fluid presented oligoclonal bands. At that time, an immunomodulatory therapy with interferon beta 1a i.m. was started. In the following 3 years, the patient suffered from four further relapses. Each time, high dose intravenous methylprednisolone pulses were applied and the patient recovered completely. After the fourth relapse, EDSS was 2.0 (see clinical course in Figure 1A). Over the following 5 years, adherence to disease-modifying therapy was poor. Consequently, the patient presented a severe relapse with paraparesis and EDSS progressed from 2.0 to EDSS 6.0 (Figure 1A). At that time, loss of her working ability as a clerk accountant was incipient and the patient was no longer able to independently care for her 9-year-old son. Thus, an escalation therapy with natalizumab was initiated which led to stabilization of the disease without further relapses and an improvement of EDSS to 2.5 (Figure 1A). After the 11th application of natalizumab, the patient developed a paresis of the right leg, which was initially considered as a relapse at an outside clinic where the patient received a high dose intravenous methylprednisolone pulse without success. Upon re-evaluation in our Department, the MRI scan of the brain showed a linear fronto-parietal cortical lesion with a very faint gadolinium enhancement (Figure 1B). A subsequently performed cerebrospinal fluid analysis revealed 30 copies of JC virus DNA and the JC virus antibody assay was positive (this test was not yet available at the beginning of therapy). The patient immediately underwent plasmapheresis therapy with five cycles for natalizumab elimination. On the basis of expert recommendations, a therapy with mirtazapine at a dosage of 60 mg and mefloquine 1,500 mg the first week and then 250 mg weekly was initiated. In the next months, repeated MRI scans after PML diagnosis of the brain presented an increase of the right fronto-parietal lesion volume with gadolinium enhancement (Figure 1B). These findings correlated with the development of a paresis oft the right hand and focal epileptic seizures of the right hand, indicating the clinical deterioration of neurological deficits during the immune reconstitution inflammatory syndrome (IRIS) after elimination of natalizumab. An antiepileptic therapy with levetiracetam was started and the patient received repeated high-dose methylprednisolone intravenously (total dosage of 20,000 mg in three cycles, see Figure 1A). Six weeks after diagnosis of PML was made, EDSS deteriorated from 4.0 to 6.5 and the patient additionally showed symptoms of major depression. Six months after the diagnosis of PML, MRI scans of the brain for the first time revealed a reduction of lesion volume and gadolinium enhancement (Figure 1B). No further focal epileptic seizures occurred and the paresis of the right hand as well as symptoms of depression completely resolved. Nine months after diagnosis of PML, the patient started to work again, although EDSS was still 5.5. The last MRI scan performed 10 months after diagnosis of PML revealed one new periventricular lesion without gadolinium enhancement. The PML lesion still showed a slight gadolinium uptake while lesion volume did no change any more (Figure 1B). So far, the patient refused to re-start any new immunomodulatory therapy.

Using flow cytometry, we analyzed the percentage of CD19 positive (+) B cells within the lymphocyte gate in active MS patients before and 3 to 6 months after initiation of natalizumab therapy (*n* = 14). Before treatment, CD19+ B cells accounted for 14.2 ± 5.5% of lymphocytes (361 ± 159 cells/μL) and increased to 21.8 ± 5.5% (876 +/− 325 cells/μL) under natalizumab therapy. In contrast, the percentage of CD19+ B cells in our splenectomized patient was 35.1% (576/μL) even before starting on natalizumab (T_0_; >3-fold SD) (Figure 1C). At the time of PML (T_1_) the percentage of CD19+ B cells within the lymphocyte gate had increased to 44.2% (approximately four-fold SD of natalizumab-treated patients; 1,303/μL) and remained high even after plasma exchange (PE) (T_2_; 52.0%, 15 days post PE; 868/μL) (Figure 1C). B cell subpopulations were further analyzed in *n* = 5 natalizumab-treated MS patients without PML. A total of 34.2 ± 11.2% of CD19+ B cells displayed a CD27+ memory phenotype which is within the range of published data on memory B cells in these patients [4]. In contrast, the percentage of CD27+ memory B cells was considerably lower in our patient both before natalizumab (T_0_; 10.5% of CD19+ cells) and at the time of PML (T_1_; 11.3% of CD19+ cells) (Figure 1D and E). Following PE, we observed a disproportional rise in CD27+ memory B cells (T_2_; 29.4% of CD19+ cells) (Figure 1D and E). Comparable observations were made for the IgM + population of memory B cells (T_0_; 10.8, T_1_; 11.5%, T_2_; 23.2%, data given as % of memory cells). In contrast, IgM + memory B cells accounted for 40.0 ± 10.9% of memory B cells in our natalizumab-treated control group. Marginal cell-like CD19+ CD27 + IgM + IgD + B cells accounted for 36.2 ± 15.2% of B cells in the natalizumab group, which is within the range of previously published data on long-term treated patients [4] (Figure 1D). In our patient, the percentage of marginal cell-like B cells tended to be lower as compared to the controls (T_0_, 20.2%; T_1_, 20.9%; T_2_, 29.0%) but was still within the two-fold of standard deviation of the controls.

## Discussion

Although the development of PML under natalizumab is extremely rare in the first year of therapy even in patients with a history of immunosuppression, this immunosuppressant-therapy naïve splenectomized MS patient developed a PML after only 11 infusions.

B cells are susceptible to JC virus infection and have been implicated in the reactivation and central nervous system (CNS) transmigration of the virus. Following the start of natalizumab therapy, the percentage of circulating B cells increases disproportionately [9,10]. A selective mobilization of B cells may thus be relevant for PML development by propagation of JCV dissemination. In addition, B cells may serve as a carrier that transports the virus to the CNS [15]. The spleen is regarded the most important reservoir of memory B cells. In addition, the spleen is the tissue that, if tested positive for JCV DNA, harbors the highest diversity of sequence rearrangements within the JCV regulatory region [9,16-18]. Splenectomy has wide-ranging consequences for immune cell homeostasis. Especially the B cell compartment seems to be affected. The percentage of CD19+ B cells among the lymphocyte population may be elevated in some but certainly not all post-splenectomy patients irrespective of the reason for splenectomy, that is trauma or hematological disease [19,20]. Yet, an increase in the percentage of circulating B cells is *per se* not considered a risk factor for infection in post-splenectomy patients. Our patient had an extraordinary high percentage of CD19+ lymphocytes within the peripheral blood that - as would be expected - even further increased under the influence of natalizumab. Given the possible role of B cells in JC virus propagation, we cannot exclude that the high pre-treatment percentage of B lymphocytes may have contributed to PML in our patient. However, the perturbed B cell memory compartment may have been the more crucial factor. Our patient had low percentages of both CD27+ and CD27 + IgM + memory B cells. This is a common finding following splenectomy [5,19]. Assessing IgM memory B cell frequency has therefore been proposed as a potential parameter for evaluation of splenic function or risk of infection following splenectomy [19,21]. Natalizumab treatment was reported to selectively mobilize memory B cells in MS patients which was considered to be secondary to an impaired retention of memory cells in the splenic marginal zone and gut-associated lymphoid tissue (GALT) by blocking very late antigen (VLA)-4/vascular cell adhesion molecule (VCAM)-1 interaction and downregulation of lymphocyte function associated antigen (LFA)-1 and alpha4-beta1 integrin [9]. In our splenectomized patient, memory B cell levels remained low under natalizumab therapy and only increased after PE. Similar observations were made for CD27 + IgM + IgD + B cells that tended to be lower in our patient. These circulating marginal zone-like B cells are considered to derive from the splenic marginal zone where these cells play an important role in the generation of T cell independent antibody responses [22]. The source for the increase in memory B cells 3 weeks following natalizumab elimination by PE in our patient is not clear but may be attributed to the GALT. We here present data of one individual only, which is a limitation of this study. In addition, our patient had been treated with glucocorticoids before diagnosis of PML was established. Since glucocorticoid treatment may affect immune cell composition of the peripheral blood, we cannot exclude that this has biased our findings. However, the profound reduction of the memory B cell population has repeatedly been demonstrated in splenectomized patients and therefore more likely to be due to the status post-splenectomy and not the glucocorticoid treatment [19]. Thus, given the prominent role of memory B cells in antiviral immune responses, splenectomy may represent a risk factor for PML under natalizumab therapy.

## Conclusions

Splenectomy may increase the risk for the development of natalizumab associated PML affecting the B cell compartment and should regarded as a risk factor in MS patients independent from the duration of disease. If therapy with natalizumab is considered in a splenectomized MS patient, benefits of the therapy should be well balanced with the possibly increased risk for the development of PML.

## Consent

The study was approved by the local ethics committee and written consent was obtained from all participants for publication of this case report and any accompanying images.

## Abbreviations

CD: Cluster of differentiation; CNS: Central nerve system; CSF: Cerebrospinal fluid; DNA: Deoxyribonucleic acid; EDSS: Expanded disability status scale; FLAIR: Flair-attenuated inversion recovery; GALT: Gut-associated lymphoid tissue; Gd: Gadolinium; HIV: Human immunodeficiency virus; IRIS: Immune reconstitution inflammatory syndrome; JCV: John Cunningham virus; LFA: Lymphocyte function associated antigen; MRI: Magnetic resonance imaging; MS: Multiple sclerosis; NK: Natural killer; PML: Progressive multifocal leukoencephalopathy; VCAM: Vascular cell adhesion molecule.

## Competing interests

The authors declare that they have no competing interest.

## Authors’ contributions

DL and AW took the lead in drafting the manuscript and have made substantial contributions to the study concept and design, analysis and interpretation of data, and statistical analysis. AW carried out FACS analyses. AL and SS were involved in drafting the manuscript, and made substantial contributions to the acquisition, analysis, and interpretation of data. AD made substantial contributions to the acquisition, analysis, and interpretation of MRI data. RL was involved in drafting the manuscript and in revising it critically for important intellectual content, and made substantial contributions to study the concept and design, and analysis and interpretation of laboratory data. All authors read and approved the final manuscript.

## Supplementary Material

Additional file 1: Figure S1 Gating strategy used to assess the frequency of CD19+ B cells, CD19 + CD27+ memory cells, IgM + memory cells and IgM + IgD + marginal cell-like B cells within the peripheral blood. Click here for file
