# Supplementary figures and images for: Immunological and clinical consequences of splenectomy in a multiple sclerosis patient treated with natalizumab
Source: J Neuroinflammation. 2013 Oct 9;10:123. doi: 10.1186/1742-2094-10-123 (PMC3854515; doi:10.1186/1742-2094-10-123)

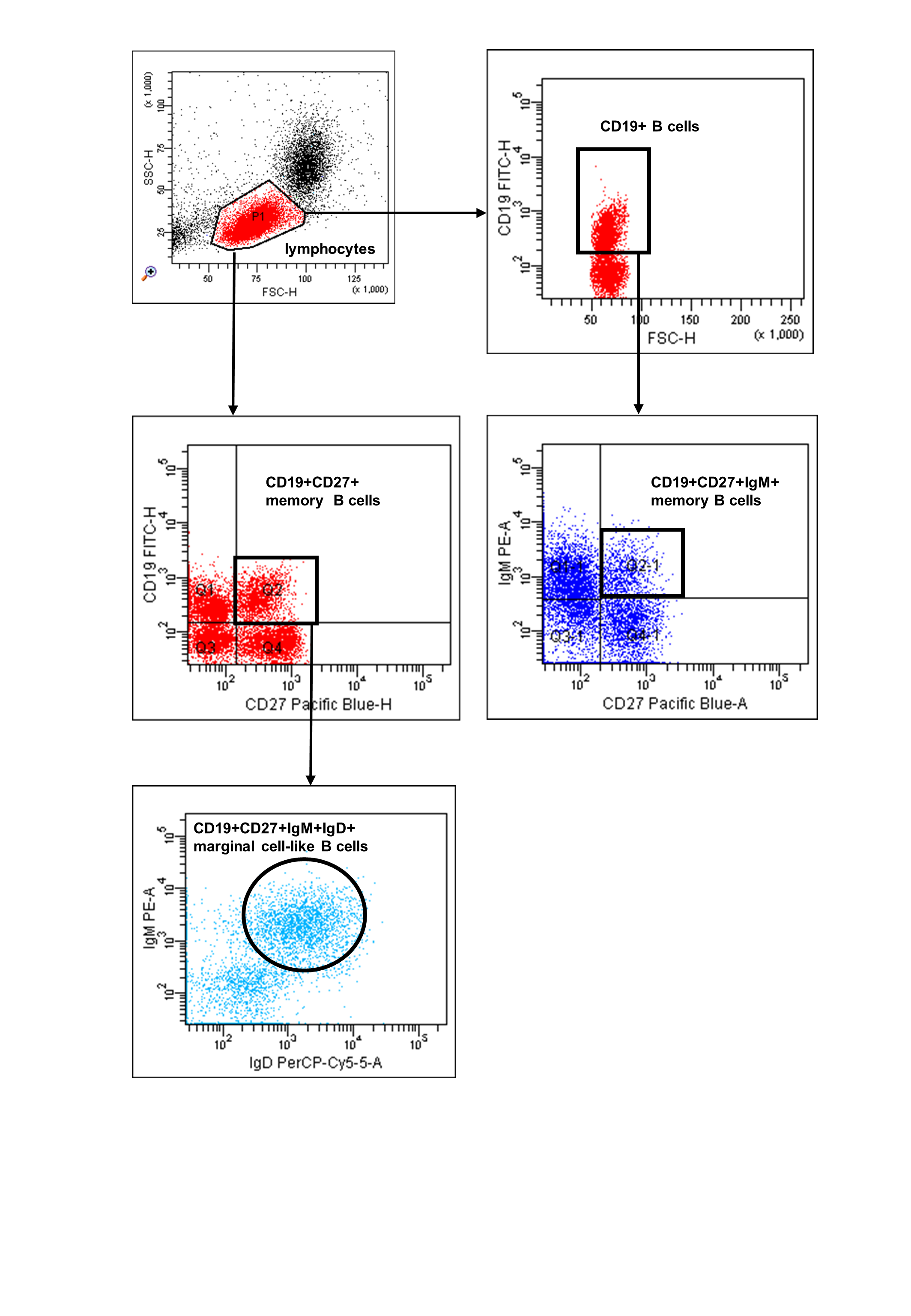

Supplement: Additional file 1: Figure S1 — Gating strategy used to assess the frequency of CD19+ B cells, CD19 + CD27+ memory cells, IgM + memory cells and IgM + IgD + marginal cell-like B cells within the peripheral blood. [file 1742-2094-10-123-S1.tiff]
